# Influence of Polyvinylpyrrolidone Molecular Weight and Concentration on the Precipitation Inhibition of Supersaturated Solutions of Poorly Soluble Drugs

**DOI:** 10.3390/pharmaceutics15061601

**Published:** 2023-05-26

**Authors:** Afnan Bany Odeh, Boushra El-Sayed, Matthias Manne Knopp, Thomas Rades, Lasse Ingerslev Blaabjerg

**Affiliations:** 1 Department of Pharmacy, University of Copenhagen, Universitetsparken 2, 2100 Copenhagen, Denmark; 2 Bioneer:FARMA, Department of Pharmacy, Universitetsparken 2, 2100 Copenhagen, Denmark; 3 Novo Nordisk, Novo Nordisk Park 2, 2760 Maaloev, Denmark

**Keywords:** supersaturation, nucleation, crystal growth, polymer, precipitation inhibitor, solvent-shift method

## Abstract

Supersaturating drug delivery systems such as solid dispersions of a drug in a polymer are frequently used in pharmaceutical development to enable oral delivery of poorly soluble drugs. In this study, the influence of the concentration and molecular weight of polyvinylpyrrolidone (PVP) on the precipitation inhibition of the poorly soluble drugs albendazole, ketoconazole and tadalafil is investigated to expand the understanding of the mechanism of PVP as a polymeric precipitation inhibitor. A three-level full-factorial design was used to delineate the influence of polymer concentration and viscosity of the dissolution medium on precipitation inhibition. Solutions of PVP K15, K30, K60 or K120 at concentrations of 0.1, 0.5 and 1% (*w*/*v*), as well as isoviscous solutions of PVP of increasing molecular weight, were prepared. Supersaturation of the three model drugs was induced by the use of a solvent-shift method. Precipitation of the three model drugs from supersaturated solutions in the absence and presence of polymer was investigated by the use of a solvent-shift method. Time–concentration profiles of the respective drugs in the absence and presence of polymer pre-dissolved in the dissolution medium were obtained by the use of a μDISS Profiler™ to determine the onset of nucleation and the precipitation rate. Multiple linear regression was used to evaluate the hypothesis that precipitation inhibition is influenced by the PVP concentration (i.e., the number of repeat units of the polymer) and the medium viscosity of the polymer for the three model drugs. This study showed that an increased concentration of PVP (i.e., an increased concentration of the PVP repeat units, independent of the molecular weight of the polymer) in solution increased the onset of nucleation and decreased the precipitation rate of the respective drugs during supersaturation, which can be explained by an increase in molecular interactions between the drug and polymer with increasing concentrations of polymer. In contrast, the medium viscosity had no significant influence on the onset of the nucleation and precipitation rate of the drugs, which can be explained by solution viscosity having a negligible effect on the rate of drug diffusion from bulk solution to the crystal nuclei. In conclusion, the precipitation inhibition of the respective drugs is influenced by the concentration of PVP, i.e., by molecular interactions between the drug and polymer. In contrast, the molecular mobility of the drug in solution, i.e., the medium viscosity, has no influence on the precipitation inhibition of the drugs.

## 1. Introduction

An increasing number of new pharmaceutical drugs exhibit poor aqueous solubility, which is likely to cause challenges during their development into oral drug products [1,2]. This has triggered the investigation of several strategies to increase the apparent solubility and dissolution rate of these poorly soluble drugs, including the use of supersaturating drug delivery systems (SDDSs) [2]. In an SDDS, the supersaturated solution of the drug exists in its thermodynamically unfavorable state, which entails precipitation through nucleation and crystal growth [3,4]. Nucleation is the formation of new crystal nuclei, and crystal growth is the growth of the formed nuclei into crystals [5,6]. A polymer is often incorporated into the SDDS to inhibit the precipitation of the drug from the supersaturated solution, by reducing the rate of nucleation and/or crystal growth of the drug [4]. Several different properties of the polymer, such as its aqueous solubility, solution viscosity and number of hydrogen bond acceptors/donors, have been suggested to affect its ability to act as a precipitation inhibitor (PI) [7,8]. Polyvinylpyrrolidone (PVP) is a polymer that is widely used as a PI because of its high aqueous solubility and as it is neither thermo- nor pH-sensitive under physiologically relevant conditions [9]. PVP is commercially available in several different molecular weights, often denoted by its K value that reflects its effect on the viscosity of aqueous solutions.

It has previously been reported that an increasing concentration of PVP in a solution is increasingly more efficient in maintaining a supersaturated solution of a drug [4,8,10]. This effect may be explained by the ability of the polymer to increase the equilibrium solubility of the drug and thereby effectively decrease the degree of supersaturation of the drug in solution. A previous study investigated the influence of different concentrations of PVP K12 on the solubility of benzthiazide in an aqueous solution of this polymer [11]. The study showed an increase in the solubility of benzthiazide with increasing concentrations of PVP K12 and an increase in the onset time of nucleation from 4 min in the absence of polymer to between 15 and 45 min with increasing concentrations of PVP K12. Additionally, the effect of a polymer in maintaining a supersaturated solution of a drug may also be explained by the ability of the polymer to form hydrogen bonds with the drug and thereby effectively decrease the self-association of the drug in solution. A previous study investigated the precipitation rate of hydrocortisone from a supersaturated solution in the presence of either hydroxypropyl methylcellulose (HPMC) or PVP [10]. HPMC contains more functional groups that can form hydrogen bonds compared to PVP and was also shown to be more effective in inhibiting the precipitation of hydrocortisone [10]. Finally, the effect of a polymer on maintaining a supersaturated solution of the drug has also been suggested to be a result of the ability of the polymer to increase the solution viscosity and thereby effectively decrease the molecular mobility of the drug in solution. A previous study investigated the precipitation rate of paracetamol from a supersaturated solution in the presence of PVP with a molecular weight of either 2000, 10,000 or 50,000 Da. The study showed that PVP 10,000 Da and PVP 50,000 Da were more effective in inhibiting the precipitation rate of paracetamol compared to PVP 2000 Da, but the study did not explain whether this effect of PVP was attributed to an increase in solution viscosity or an increase in molecular interactions between the drug and PVP [8]. However, in solutions with the same concentration of PVP, the number of potential binding sites remains the same, irrespective of the molecular weight of PVP, i.e., the same amount of repeating monomer units, which could indicate that the effect of PVP can be explained by the increased solution viscosity with the higher-molecular-weight PVP. The ability of PVP to affect the onset of the nucleation and precipitation rate of a drug in a supersaturated solution is therefore not fully elucidated, which could be because it is drug dependent [12]. The purpose of this study is to expand the current knowledge on the influence of PVP as a PI on the onset of nucleation and rate of precipitation of a drug in a supersaturated solution. Three different poorly soluble drugs representing the class II of the Biopharmaceutics Classification System, albendazole, tadalafil and ketoconazole, were selected due to their presence or absence of hydrogen bond donors to delineate the influence of potential drug–polymer interactions on their respective precipitation from supersaturated solutions. Furthermore, different molecular weights and concentrations of PVP were investigated to delineate the influence of dissolution medium viscosity and potential drug–polymer molecular interactions on the rate of precipitation of the model drugs from supersaturated solutions. The effective concentration of polymer on the precipitation inhibition of a drug during supersaturation has been shown to be as low as 0.001% (*w*/*v*) in several studies [13,14]. For selecting appropriate polymer concentrations, a more pragmatic approach is to assume that a solid dosage form of 500 mg containing 20% (*w*/*w*) polymer dispersed in the volume of the small intestine in the fasted state (105 ± 72 mL) [15] will result in polymer concentrations in vivo between 0.1% and 1.0% (*w*/*v*).

The inhibition of the nucleation and precipitation rate of the respective drugs, in the absence and presence of PVP, were investigated by the use of a solvent-shift method [7]. The pH-shift or solvent-shift methods are commonly used to induce supersaturation of a drug, i.e., by using the drug in a pre-dissolved form. The pH-shift method is only suitable for ionizable drugs as supersaturation of the drug is induced by dissolving the drug in a solution where it is ionized and then titrating the solution to obtain its neutral form. In the solvent-shift method, supersaturation of the drug is induced by diluting a solution of highly concentrated drug in a suitable solvent into a solution in which the drug has a low solubility. Finally, supersaturation of the drug can also be induced by powder dissolution of an SDDS of the drug. In contrast to powder dissolution and the pH-shift method, the solvent-shift method is uncomplicated to use and only requires that the two solutions used are miscible.

The induction time of the time–concentration profiles is used as a surrogate for the inhibition of the nucleation of the respective drugs, and the slope of the time–concentration profiles is used as a surrogate for the precipitation rate of the respective drugs.

## 2. Materials and Methods

### 2.1. Materials

**Figure 1:**
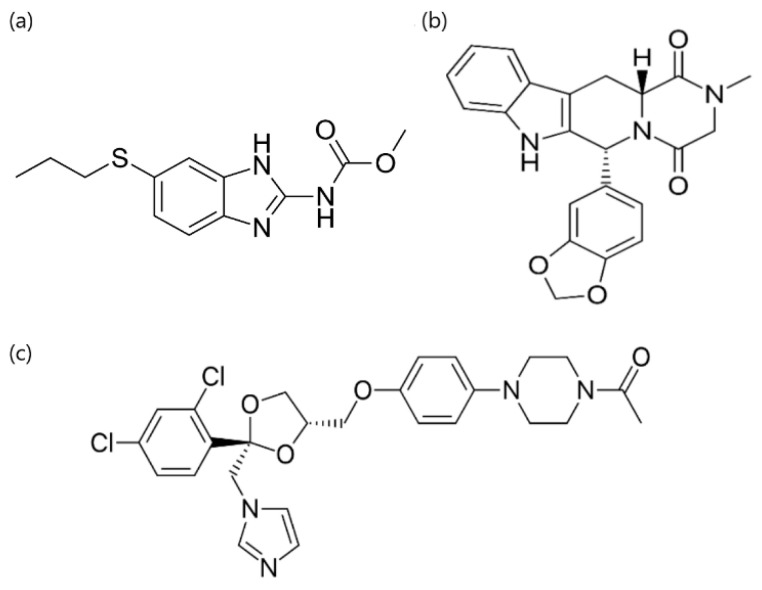
Molecular structures of (**a**) albendazole, (**b**) tadalafil and (**c**) ketoconazole.

Albendazole (Figure 1) was acquired from Sigma-Aldrich (Shanghai, China), tadalafil (Figure 1) from AK Scientific (Union City, CA, USA) and ketoconazole (Figure 1) from Fagron Nordic (Copenhagen, Denmark). Polyvinylpyrrolidone K15, K30 and K60 were acquired from Sigma-Aldrich (Shanghai, China) and polyvinylpyrrolidone K120 from Ashland (Texas City, TX, USA). Di-sodium hydrogen phosphate dihydrate, sodium dihydrogen phosphate monohydrate, hydrochloric acid (HCl), sodium hydroxide (NaOH), di-ammonium hydrogen phosphate, sodium sulfate, dimethyl sulfoxide (DMSO) and acetonitrile were all acquired from Merck (Darmstadt, Germany). Ammonium phosphate and phosphoric acid were acquired from Sigma-Aldrich (Buchs, Switzerland). The materials were used as received.

### 2.2. Preparation of PVP Solutions

Stock solutions of 1% (*w*/*v*) PVP K15, K30, K60 and K120 were prepared in 0.1 M phosphate buffer adjusted to pH 7.4. The stock solutions were diluted with 0.1 M phosphate buffer pH 7.4 to concentrations of 0.5% and 0.1% (*w*/*v*). Isoviscous solutions of PVP (corresponding to a viscosity of 1 mPa s) were prepared at concentrations of 3.13% (*w*/*v*) K15, 2.81% (*w*/*v*) K30, 0.33% (*w*/*v*) K60 and 0.25% (*w*/*v*) K120 in 0.1 M phosphate buffer pH 7.4. The moisture content of the polymer was considered in the preparation of all solutions of PVP.

### 2.3. Thermogravimetric Analysis of PVP

Thermogravimetric analysis of PVP was used to measure the moisture content of PVP K15, K30, K60 and K120 prior to use, using a TA Instruments Discovery TGA (New Castle, DE, USA). A 5–10 mg amount of sample was weighed into a platinum pan, and the sample was then subjected to heating from 25 to 110 °C using a rate of 10 °C/min. The sample oven was purged with nitrogen at a flow rate of 50 mL/min. TA Instruments Trios software (New Castle, DE, USA) was used to analyze the resulting temperature–weight diagrams, by calculating the weight loss between 25 and 110 °C. Measurements were performed in triplicate. The moisture contents for PVP K15, K30, K60 and K120 were measured to be 6.6% *w*/*w* ± 0.6, 6.6% *w*/*w* ± 1.3, 3.8% *w*/*w* ± 0.5 and 6.9% *w*/*w* ± 0.1, respectively (mean ± SD).

### 2.4. Equilibrium Solubility of the Drugs in PVP Solutions

The equilibrium solubility of albendazole, ketoconazole and tadalafil was determined by a shake-flask solubility test in the absence and presence of PVP. An excess amount of the drug was added to 15 mL corning tubes containing either pure 0.1 M phosphate buffer (pH 7.4) or 0.1, 0.5 and 1% (*w*/*v*) solutions of PVP K15, K30, K60 or K120, respectively. The samples were then capped and stirred overnight in an oven at 37 °C.

The saturated solution and the precipitate were separated by centrifugation for 10 min at 13,000 rpm in an Eppendorf Centrifuge 5810 R (Hamburg, Germany), followed by filtration intro ultra-performance liquid chromatography (UPLC) vials using a syringe filter (33 mm, 0.45 µm) PVDF membrane (2 mL filter sample prewet volume), acquired from Merck (Darmstadt, Germany). The tests were conducted in triplicate.

### 2.5. Quantitative Analysis

The concentration of drug in the respective samples was quantified by reverse-phase chromatography using a Waters Acquity Classic UPLC system (Milford, MA, USA) equipped with an automated sample injector, a binary pump and a tunable UV detector. All samples were analyzed using a Waters Acquity UPLC BEH C18 column (130 Å, 1.7 µm, 2.1 × 50 mm) (Milford, MA, USA) at a column temperature of 50 °C for ketoconazole and tadalafil and 80 °C for albendazole. The samples were injected with a volume of s 6 µL and subsequently eluted at a flow rate of 0.8 mL/min. For albendazole and tadalafil, eluent A was a mixture of 90% (*v*/*v*) sodium sulfate (60 mM), di-ammonium hydrogen phosphate (5.5 mM) and phosphoric acid (34.5 mM) in Milli-Q water (pH 2.3) and 10% (*v*/*v*) acetonitrile. For ketoconazole, eluent A was a mixture of 90% (*v*/*v*) sodium sulfate (60 mM), di-ammonium hydrogen phosphate (5.5 mM) and phosphoric acid (34.5 mM) in Milli-Q water (pH 7.2) and 10% (*v*/*v*) acetonitrile. For tadalafil, albendazole and ketoconazole, eluent B was a mixture of 20% (*v*/*v*) Milli-Q water and 80% (*v*/*v*) acetonitrile. Albendazole was quantified in the samples using UV at a wavelength of 229 nm, while tadalafil was quantified at 284 nm and ketoconazole at 240 nm. Standard curves were prepared in the range of 0.1–0.8 µg/mL for albendazole, 0.6–12.0 µg/mL for tadalafil and 1–4 µg/mL for ketoconazole (R^2^ > 0.98) (Appendix A), and samples were analyzed using Waters Empower Software (version 3.0 build 3471, Milford, MA, USA).

### 2.6. Viscosity of Solutions of PVP

The viscosities of the PVP solutions were determined to delineate the influence of the viscosity and potential drug–polymer molecular interactions on the onset of the nucleation and precipitation rate of the respective drugs from supersaturated solutions. Determination of the viscosity of the respective solutions of PVP was conducted using the cone and plate geometry technique on a TA Instruments AR2000 rheometer (New Castle, DE, USA) mounted with a 60 mm stainless-steel cone with an angle of 1° (truncation gap 29 µm). Shear rates of 1–1300 s^−1^ were investigated using 5 points per decade. The pressure was adjusted to 2 bars, and the measurements were carried out at 37 °C. The experiments were performed in triplicates with a sample volume of about 1–2 mL. Microsoft Excel (Redmond, WA, USA) was used for data analysis. Based on the behavior of the PVP solutions as non-Newtonian fluids, a shear rate at the constant viscosity plateau at 631 s^−1^ was chosen to determine the solution viscosity (Appendix B and Appendix C).

### 2.7. Nucleation and Crystal Growth of Drugs in a Supersaturated Solution

Time–concentration profiles of the drugs in a supersaturated solution in the absence and presence of PVP were obtained to study the influence of the polymer on the onset of the nucleation and precipitation rate of the drug from a supersaturated solution. Supersaturation of the drug was induced by the use of a method previously developed [7]. Briefly, a PION µDISS Profiler™ (Billerica, MA, USA) was used to obtain in situ UV spectra in the range from 200 to 720 nm. Probe tip lengths of 5, 10 or 20 mm were selected to obtain an absorbance in the range between 0.3 and 1. The vials were stirred using a crossbar magnet at ~100 rpm at a temperature of 37 °C. The probe tip length was found to be 20 mm for albendazole, 20 mm for ketoconazole and 5 mm for tadalafil. The UV wavelength range used to quantify the drug was 267–288 nm for albendazole, 304–360 nm for ketoconazole and 299–328 nm for tadalafil. Standard curves were prepared with a concentration range of 0–12 µg/mL for albendazole, 0–70 µg/mL for tadalafil and 0–90 µg/mL for ketoconazole (R^2^ = 0.999). Standard curves were prepared with six replicates by adding aliquots of 15 µL DMSO stock solution of the drug to the dissolution medium. The initial concentration of the drug was determined by using the highest concentration of the drug for which no instantaneous precipitation occurred. Precipitation was detected by a deviation from the linearity of the standard curve, a sharp shift in the baseline in the UV spectrum or through visual inspection of the solution. To induce supersaturation, 100 µL of a DMSO stock solution of the drug was transferred into glass vials containing 10 mL solutions of PVP K15, K30, K60 or K120. The concentrations of albendazole, ketoconazole and tadalafil in the DMSO stock solutions were 1, 8 and 6 mg/mL, respectively.

The drug concentration was monitored in situ by the second derivative of the UV absorbance. The second derivative was used to minimize UV interferences from the precipitate. The experiments were ended when the concentrations of the respective drugs reached their equilibrium solubility.

### 2.8. Data Analysis

The induction time of the time–concentration profiles was used as a surrogate for the onset of the nucleation of the respective drugs, and the slope of the time–concentration profiles was used as a surrogate for the precipitation rate of the respective drugs. The induction time (t_ind_) and the slope of the time–concentration profile were determined using TA Instruments Trios software (New Castle, DE, USA). The induction time of the time–concentration profile in minutes was estimated using the onset time function via the tangent. The slope of the time–concentration profile in (µg/mL)/min was estimated using the slope function.

### 2.9. Statistical Analysis

Multiple linear regression analysis was conducted using Umetrics MODDE11 pro (Glostrup, Denmark) to delineate the influence of PVP molecular weight and concentration on the onset of nucleation and the precipitation rate of albendazole, tadalafil and ketoconazole. The t_ind_ and slope from the time–concentration profiles were used as the response values, whereas PVP concentration and the viscosity of the PVP solutions were used as the input predictors. It should be noted that the viscosity of the PVP solutions is used instead of the molecular weights of PVP, and therefore, the impact of the viscosity of solutions of PVP is the same as that of the molecular weight. The three levels for the concentration factor were 0.1, 0.5 and 1% (*w*/*v*), and the four levels for the viscosity factor were the viscosities of the solutions of PVP K15, K30, K60 and K120. The multiple linear regression coefficient plot displayed regression coefficients with 95% confidence interval. A factor was considered to have a significant influence if the standard deviation did not cross zero.

The equilibrium solubility, viscosity of the PVP solutions, t_ind_ and slope of time–concentration profile during supersaturation of the drugs are given as the mean ± standard deviation (SD). To assess statistically significant differences between groups, the obtained results were subjected to a one-way analysis of variance (ANOVA), followed by Tukey’s pairwise comparison. Microsoft Excel (Redmond, WA, USA) was used for this analysis. The criteria for determining statistical significance in all tests were set at *p* < 0.05.

## 3. Results and Discussion

### 3.1. Influence of PVP Concentration and Molecular Weight on the Solubility of the Drugs

The equilibrium solubility of albendazole, tadalafil and ketoconazole in solutions of 0.1, 0.5 and 1% (*w*/*v*) PVP K15, K30, K60 or K120, respectively, was determined to delineate the influence of PVP concentration and molecular weight on the equilibrium solubility of the drugs. The equilibrium solubility of the respective drugs was obtained using the shake-flask solubility test.

**Figure 2:**
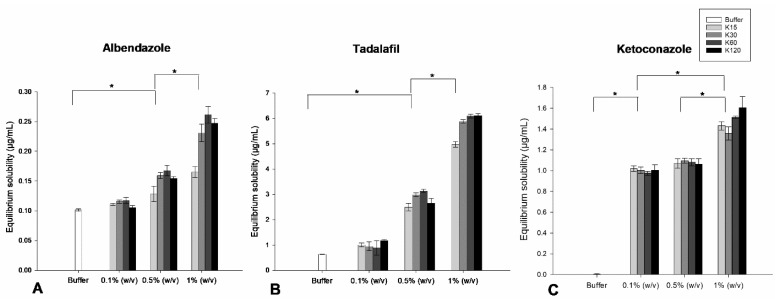
Equilibrium solubility of albendazole (**A**), tadalafil (**B**) and ketoconazole (**C**) in absence and presence of solutions of 0.1, 0.5 and 1% (*w*/*v*) PVP K15, K30, K60 and K120 (*n* = 3) (mean ± SD). Asterisk (*) indicating statistically significant differences (*p* < 0.05).

ANOVA showed no statistically significant increase in the equilibrium solubility of albendazole and tadalafil in the solution of 0.1% (*w*/*v*) PVP compared to the phosphate buffer. This result is in line with a previous study that investigated the equilibrium solubility of albendazole and tadalafil in the presence of 0.1% (*w*/*v*) pre-dispersed PVP K30 [16]. The study showed no significant increase in the equilibrium solubility of the drugs in the presence of 0.1 (*w*/*v*) PVP K30 [16]. However, a statistically significant increase (*p* < 0.05) in the equilibrium solubility of albendazole and tadalafil was observed in solutions of 0.5 and 1% (*w*/*v*) PVP, respectively (Figure 2A,B). This is also in line with another study that investigated the equilibrium solubility of albendazole in the presence of different polymers and showed an increase in the equilibrium solubility of albendazole in the presence of 0.2% pre-dissolved PVP K30 [17].

A statistically significant increase (*p* < 0.05) in the equilibrium solubility of ketoconazole in solutions of 0.1% and 0.5% (*w*/*v*) PVP compared to the equilibrium solubility in phosphate buffer was found, with a further statistically significant increase (*p* < 0.05) in the equilibrium solubility of ketoconazole when increasing the concentration of PVP to 1% (*w*/*v*) (Figure 2C). These results are in alignment with a previous study that showed an increased equilibrium solubility of ketoconazole in phosphate buffer in the presence of pre-dissolved PVP K30 and a further increase with increasing concentrations of 1 to 8% (*w*/*v*) PVP K30 [18].

No statistically significant increase (*p* > 0.05) in the equilibrium solubility of albendazole, tadalafil and ketoconazole was observed when having the same concentration of repeating monomer units of PVP K15, K30, K60 and K120 (Figure 2). This could be explained by the number of potential binding sites of PVP being the same, irrespective of the molecular weight in the same concentration. Albendazole and tadalafil both contain hydrogen bond donors to potentially form hydrogen bonds with PVP. It is well known that an increase in repeating monomeric units of PVP leads to more hydrogen bond acceptors from the N-vinylpyrrolidone group of PVP and thereby increased molecular interaction between drug and polymer, leading to a potentially higher solubility of the drug [4]. In contrast, ketoconazole does not have any hydrogen bond donor groups to interact with PVP. However, a previous study investigated specific drug–polymer interactions between ketoconazole and PVP. Here, infrared spectroscopy showed that ketoconazole and PVP can interact by forming weak dipole–dipole interactions, and it is therefore speculated that these interactions can lead to an increase in the solubility of the drug [19].

### 3.2. Influence of PVP Concentration and Molecular Weight on Solution Viscosity

**Figure 3:**
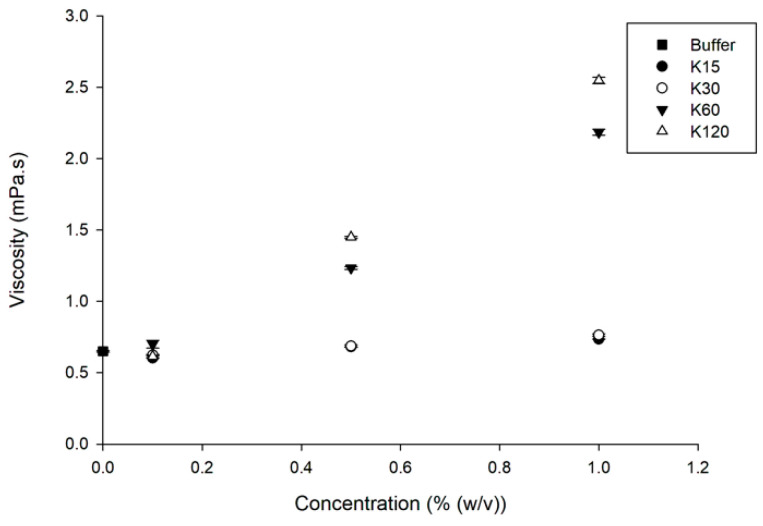
Viscosities of the solutions of PVP K15, K30, K60 and K120 at concentrations of 0.1, 0.5 and 1% (*w*/*v*) at a shear rate of 631 s^−1^ (*n* = 3) (mean ± SD).

The viscosities of solutions of PVP K15, K30, K60 and K120 were determined to delineate the influence of the dissolution medium viscosity and potential drug–polymer molecular interactions on the precipitation rate of the drug from a supersaturated solution (Figure 3).

ANOVA showed no statistically significant difference (*p* > 0.05) in solution viscosity for water, PVP K15 and K30 in the concentration range of 0.1 to 1% (*w*/*v*) PVP. In contrast, the solution viscosities for PVP K60 and K120 were statistically significantly different (*p* < 0.05) and increased with increasing concentration. As expected, PVP K120 had the highest solution viscosity for all concentrations of PVP, compared to PVP K15, K30 and K60.

Several studies have claimed that the precipitation rate of a drug from a supersaturated solution can be slowed down by the use of a polymer to effectively increase solution viscosity and thereby decrease the molecular mobility of the drug in solution to the crystal nuclei [10,11].

To further investigate the relationship between the solution viscosity and precipitation rate of the drug, supersaturation studies of the respective drugs in isoviscous solutions were conducted. To prepare isoviscous solutions of PVP, the corresponding concentration of the respective polymers were obtained from linear regression of the viscosities obtained for solutions 0.1, 0.5 and 1% (*w*/*v*) PVP. The data indicated that PVP K15, K30, K60 and K120 at concentrations of 3.13, 2.81, 0.33 and 0.25% (*w*/*v*), respectively, would result in a viscosity of 1 mPa.s. ANOVA showed no statistically significant difference (*p* > 0.05) in the viscosities of the isoviscous solutions of PVP (Table 1).

### 3.3. Supersaturation Studies

The precipitation inhibition of albendazole, tadalafil and ketoconazole in the absence and presence of solutions of PVP was investigated using a µDISS Profiler. The induction time (t_ind_) of the time–concentration profiles of the respective drugs was used as a surrogate for the onset of nucleation and the slope of the time–concentration profiles was used as a surrogate for the precipitation rate of the respective drugs (Appendix D).

#### 3.3.1. Influence of PVP Concentration, Solution Viscosity and Molecular Weight on the t_ind_ of the Drugs

**Figure 4:**
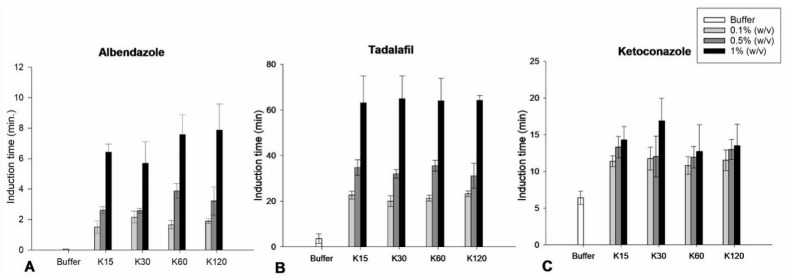
Induction time (t_ind_) of the time–concentration profiles of albendazole (**A**), tadalafil (**B**) and (**C**) ketoconazole in absence and presence of aqueous solutions of PVP K15, K30, K60 and K120 at concentrations of 0.1, 0.5 and 1% (*w*/*v*) (*n* = 8) (mean ± SD).

For all drugs, the t_ind_ generally increased with increasing concentrations of PVP K15, K30, K60 and K120 from 0.1 to 0.5% (*w*/*v*) and again from 0.5 to 1% (*w*/*v*) compared to the t_ind_ in the absence of PVP (Figure 4).

In solutions of PVP K15, K30, K60 and K120, ANOVA showed a statistically significant increase (*p* < 0.05) of t_ind_ of albendazole and tadalafil when increasing the PVP concentration from 0.1 to 0.5% (*w*/*v*) and again from 0.5 to 1% (*w*/*v*), respectively (Figure 4A,B).

In a solution of PVP K15, ANOVA showed a statistically significant increase (*p* < 0.05) in the t_ind_ of ketoconazole when increasing the PVP concentrations from 0.1 to 0.5% (*w*/*v*) and again from 0.5 to 1% (*w*/*v*) (Figure 4C). In contrast, in a solution of PVP K30, no statistically significant increase (*p* > 0.05) in the t_ind_ of ketoconazole was found when increasing the PVP concentration from 0.1 to 0.5% (*w*/*v*). However, ANOVA showed a statistically significant increase (*p* < 0.05) in the t_ind_ of ketoconazole when increasing the PVP concentration from 0.5 to 1% (*w*/*v*). Additionally, in solutions of PVP K60 and K120, ANOVA showed no statistically significant differences (*p* > 0.05) of the t_ind_ of ketoconazole when increasing the PVP concentration from 0.1 to 0.5% (*w*/*v*) and again from 0.5 to 1% (*w*/*v*).

The positive correlation between the onset of nucleation and the concentration of the polymer can be explained by the increase in available functional groups in the polymer, resulting in an increase in drug–polymer interaction [4]. The increase in the onset of nucleation may also be explained by the increase in polymer concentration effectively decreasing the degree of supersaturation. These results are in line with a previous study that investigated the influence of different polymers on the supersaturation of different compounds at different degrees of supersaturation using the µDISS Profiler. The study observed an increase in the t_ind_ of albendazole and tadalafil when decreasing the degree of supersaturation of the drugs [7]. In contrast, a previous study investigated the influence of PVP K30 on the onset of the nucleation of bicalutamide from a supersaturated solution and found no significant effect of PVP. It was speculated that this could be explained by the critical nucleus size being slightly larger than the size of the repeating monomer unit of PVP [20].

**Figure 5:**
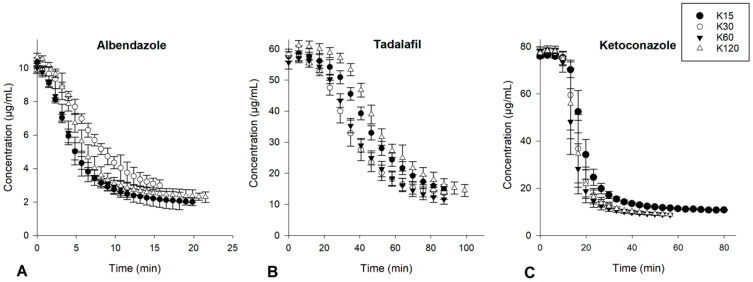
Time–concentration profiles of albendazole (**A**), tadalafil (**B**) and ketoconazole (**C**) in aqueous solutions PVP K15, K30, K60 and K120 at a concentration of 0.1% (*w*/*v*) (*n* = 8) (mean ± SD).

**Figure A5:**
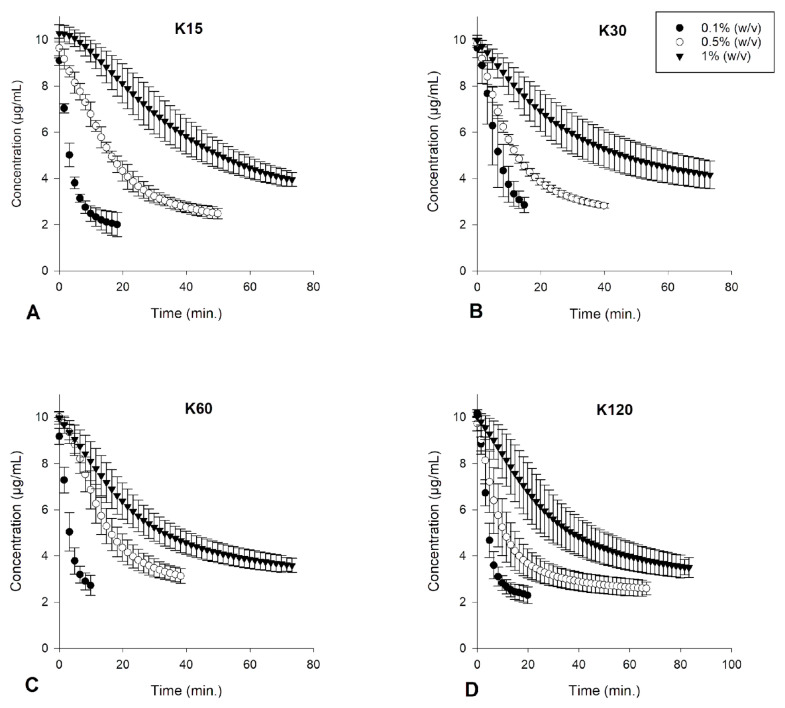
Time–concentration profiles of supersaturated solutions of albendazole in solutions of PVP K15 (**A**), K30 (**B**), K60 (**C**) and K120 (**D**) at the concentrations of 0.1, 0.5 and 1% (*w*/*v*) (*n* = 8) (mean ± SD).

**Figure A6:**
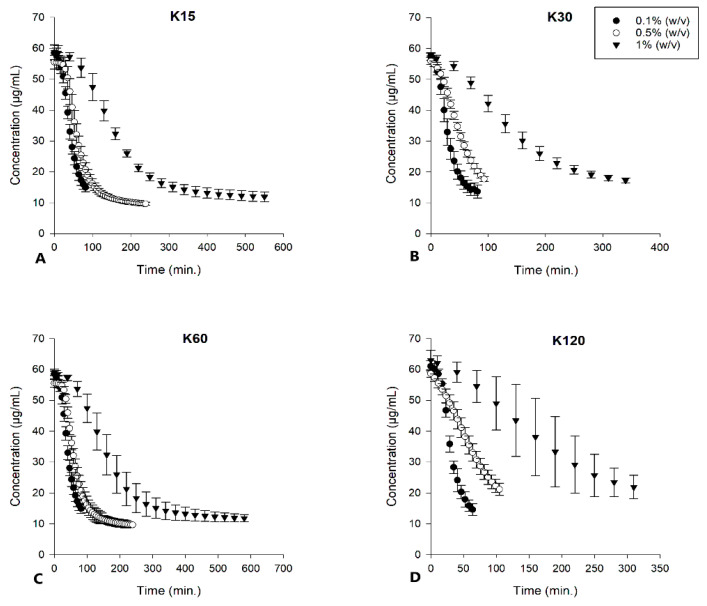
Time–concentration profiles of supersaturated solutions of tadalafil in solutions of PVP K15 (**A**), K30 (**B**), K60 (**C**) and K120 (**D**) at the concentrations of 0.1, 0.5 and 1% (*w*/*v*) (*n* = 8) (mean ± SD).

**Figure A7:**
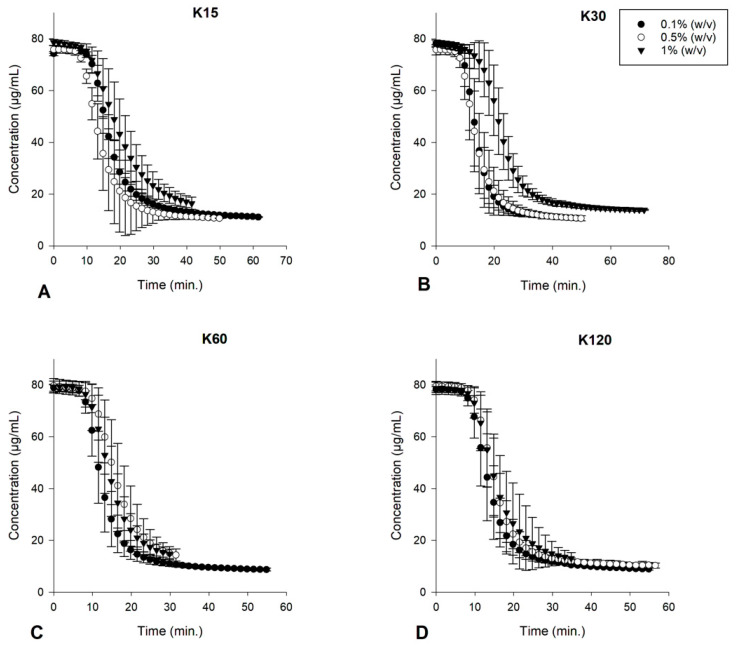
Time–concentration profiles of supersaturated solutions of ketoconazole in solutions of PVP K15 (**A**), K30 (**B**), K60 (**C**) and K120 (**D**) at the concentrations of 0.1, 0.5 and 1% (*w*/*v*) (*n* = 8) (mean ± SD).

For albendazole, tadalafil and ketoconazole, ANOVA showed no statistically significant differences (*p* > 0.05) in the t_ind_ in the presence of the same concentrations of PVP K15, K30, K60 and K120 at concentrations of 0.1, 0.5 and 1% (*w*/*v*) as can be seen in Figure 5 for 0.1% (*w*/*v*) PVP and Figure A5, Figure A6 and Figure A7 for 0.5 and 1% (*w*/*v*) PVP. This can be explained by the number of potential binding sites of PVP staying the same, irrespective of the PVP molecular weight, i.e., the same amount of repeating monomer units when using the same concentration [21]. This means that the molecular weight of PVP has no significant influence on the onset of nucleation and, consequently, also that the viscosity of the PVP solution has no influence on the onset of the nucleation of the drug from a supersaturated solution. These results are in line with a previous study that showed no correlation between the solution viscosity and the t_ind_ of supersaturated nifedipine in solutions of either 0.05% (*w*/*v*) PVP K17, K25 or K30 or 0.05% HPMC E3, E5, E6, E15 or E50 [22].

#### 3.3.2. Influence of PVP Concentration, Solution Viscosity and Molecular Weight on the Precipitation Rate of the Drugs

**Figure 6:**
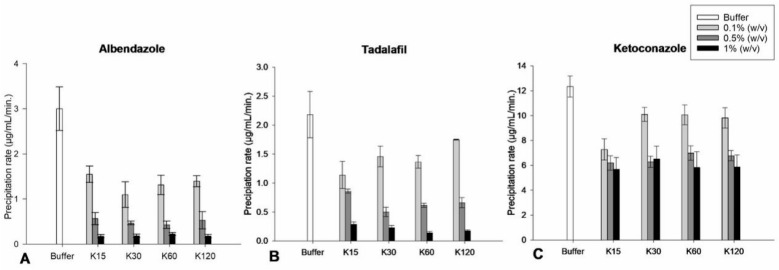
Precipitation rates of the time–concentration profiles of albendazole (**A**), tadalafil (**B**) and ketoconazole (**C**) in absence and presence of solutions of PVP K15, K30, K60 and K120 at concentrations of 0.1, 0.5 and 1% (*w*/*v*) (*n* = 8) (mean ± SD).

For albendazole, tadalafil and ketoconazole, the slope of the time–concentration profile generally decreased with increasing concentrations of PVP K15, K30, K60 and K120 in solutions compared to the slope of the time–concentration profiles in the absence of PVP (Figure 6). For albendazole and tadalafil (Figure 6A,B), ANOVA showed a statistically significant difference (*p* < 0.05) in the slope of the time–concentration profile of the drugs in the presence of solutions of PVP K15, K30, K60 and K120 when increasing the PVP concentration from 0.1 to 0.5% (*w*/*v*) and again from 0.5 to 1% (*w*/*v*). This can be explained by the solubility of the drug increasing with increasing concentrations of PVP and thereby effectively decreasing the degree of supersaturation, leading to a slower precipitation rate [3,4,6]. This result is in line with a previous study that investigated the precipitation rate of indomethacin from a supersaturated solution at degrees of supersaturation of <1.6, 2 and 6 and found that increasing the degree of supersaturation resulted in an increased precipitation rate [14]. Furthermore, another study conducting a similar investigation showed that at a high degree of supersaturation of indomethacin in the absence of PVP, the precipitation rate was mainly diffusion controlled; however, in the presence of PVP, the precipitation rate was slowed down as the mechanism shifted to surface integration being controlled by the polymer providing an interfacial barrier for crystal growth by hydrogen bonding to indomethacin [23].

For albendazole and tadalafil, ANOVA showed no statistically significant differences (*p* > 0.05) in the slope of the time–concentration profiles in the presence of PVP K15, K30, K60 and K120 at concentrations of 0.1, 0.5 and 1% (*w*/*v*), i.e., at the same amount of repeating monomer units (Figure 6A,B). In contrast, for ketoconazole (Figure 6C), ANOVA showed a statistically significant difference (*p* < 0.05) in the slope of the time–concentration profile in the presence of solutions of PVP K15, K30, K60 and K120 when increasing the concentration from 0.1 to 0.5% (*w*/*v*), but not when increasing the concentration from 0.5 to 1% (*w*/*v*). These results are in line with a previous study that investigated the influence of polymer molecular weight on the dissolution and precipitation rate of celecoxib in amorphous solid dispersions (ASD) [15]. The study showed that the precipitation rate decreased for an ASD with PVP K17 compared to the pure amorphous celecoxib, and that a further decrease was observed for an ASD with PVP K30. However, there was no further decrease in precipitation rate for an ASD with PVP K60 compared to PVP K30 [15].

For ketoconazole, ANOVA showed a statistically significant difference (*p* < 0.05) in the slope of the time–concentration profile of 0.1% (*w*/*v*) PVP K15 compared with PVP K30, K60 and K120, whereas the order in which PVP led to a decrease in the slope of the time–concentration profile is given as PVP K15 < K30 = K60 = K120. These results are in line with a previous study that indicated an increase in the molecular weight of PVP does not necessarily correlate with a decrease in the precipitation rate of the drug [24]. The study showed a decreasing rate of precipitation of salbutamol when adding PVP in the order of molecular weight of K40 > K25 > K10 > K360 [24]. Another study showed that 0.001% (*w*/*v*) PVP K30 resulted in a larger decrease in the precipitation rate of sulfisoxazole compared to both PVP K17 and K90 at the same concentration and suggested that the mechanism of PVP in inhibiting the precipitation of the drug was molecular interactions rather than the increase in molecular weight and thereby viscosity of the solution of PVP [25]. The results from the current study are in line with several studies that suggested that increased solution viscosity does not correlate with a decreased precipitation rate of drugs [8,12,20]. This was also observed in a later study, which evaluated solution viscosity on the precipitation inhibition of a drug by use of the co-polymer Pluronic [26]. Here, it was observed that increasing the viscosity of a solution played a negligible role in the precipitation rate of the drug.

#### 3.3.3. Multivariate Evaluation of the Influence of Concentration and Solution Viscosity on t_ind_ and Precipitation Rate of the Drugs

A coefficient plot was generated to provide an evaluation of the influence of PVP concentration and solution viscosity on the t_ind_ and precipitation rate of the drugs from supersaturated solutions.

**Figure 7:**
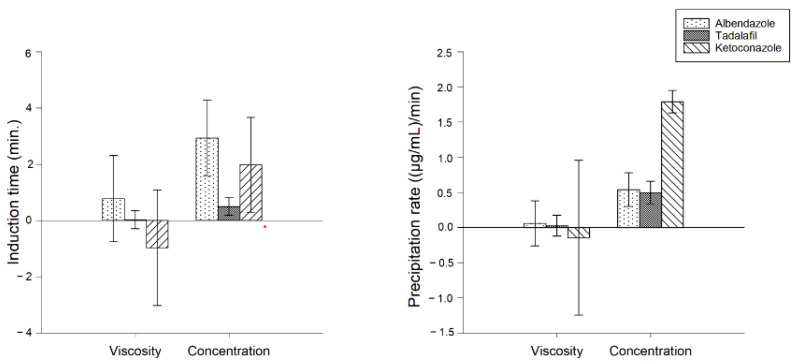
Coefficient plot generated from MODDE11 pro showing the influence of solution viscosity and PVP concentration on the t_ind_ and precipitation rate of albendazole, tadalafil and ketoconazole.

For albendazole, tadalafil and ketoconazole, the coefficient plot shows that the PVP concentration has a significant influence on the t_ind_ and slope of the time–concentration profile, while the viscosity of the PVP solution has no significant influence on either t_ind_ or slope of time–concentration profile (Figure 7).

To validate the relationship between the solution viscosities and the slope of the time–concentration profiles of the drugs, supersaturation studies of the drugs in isoviscous solutions were investigated. The precipitation inhibition of albendazole, tadalafil and ketoconazole in the presence of 1 mPa.s isoviscous solutions of PVP K15, K30, K60 or K120 was investigated, respectively.

**Figure 8:**
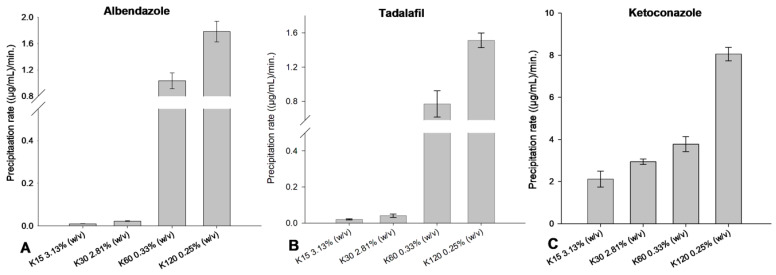
Precipitation rates of the time–concentration profiles of albendazole (**A**), tadalafil (**B**) and ketoconazole (**C**) in ~1 mPa.s isoviscous solutions of PVP K15, K30, K60 and K120 (*n* = 8) mean ± SD.

For albendazole, tadalafil and ketoconazole, ANOVA showed a statistically significant difference (*p* < 0.05) in the slope of the time–concentration profiles of the drugs in the presence of isoviscous solutions of PVP K15, K30, K60 and K120 when increasing the PVP molecular weight (Figure 8).

This indicates that there is no correlation between the precipitation rates of the respective drugs and the viscosities of solutions of PVP, as they have comparable solution viscosities but still result in different precipitation rates of the respective drugs. In turn, there is a correlation between the precipitation rate of the respective drugs and the concentration of PVP in solution, which can be explained by an increase in molecular interactions between the drug and polymer with increasing concentrations of polymer.

This study has delineated the influence of PVP concentration and viscosity on the precipitation inhibition of albendazole, tadalafil and ketoconazole. The results from the current study and several studies in the literature suggest that the viscosity of a solution has negligible influence on the precipitation rate of a drug in solution, which is in contrast to a few other studies in the literature that claim that increased solution viscosity will decrease the rate of drug diffusion from bulk solution to the crystal nuclei, leading to a slower precipitation rate [10,11]. Given that the dissolution rate of a polymer is often inversely correlated with its molecular weight, the findings in this study indicate that PVP with a low molecular weight is preferred over a high molecular weight when developing an SDDS such as an ASD. However, this effect has to be balanced with the fact that ASDs are thermodynamically unstable, and several studies have shown that an ASD containing a polymer with a high molecular weight often has longer physical stability compared to the ASD containing a lower molecular weight of the same polymer [15]. Using the same approach as in this study, future studies should therefore delineate the influence of concentration and solution viscosity for other polymers, such as HPMC and HPC to determine the applicability of the findings in the current study to other commonly used precipitation inhibitors.

## 4. Conclusions

In this study, the influence of the concentration and viscosity of solutions of PVP in buffer, on the onset of the nucleation and precipitation rate of three drugs were evaluated. Multiple linear regression was used to statistically evaluate the influence of PVP concentration and the viscosity of solutions of PVP on the precipitation of the drugs. To further investigate the relationship between the solution viscosities and precipitation rates of the drugs, supersaturation of the respective drugs was also studied in isoviscous solutions of PVP with increasing molecular weights. As expected, an increase in PVP concentration showed an increase in the t_ind_ and a decrease in the slope of the time–concentration profiles of albendazole, tadalafil and ketoconazole. However, the t_ind_ for ketoconazole in the presence of solutions of PVP K60 and K120 did not increase when increasing the PVP concentration from 0.1 to 0.5% (*w*/*v*) and again from 0.5 to 1% (*w*/*v*). These findings can be explained by drug–polymer interactions such as hydrogen bonds or dipole–dipole forces leading to an increase in drug solubility and thereby a reduction in the degree of supersaturation that in turn increases the t_ind_ and decreases t_ind_ precipitation rate of the drugs. Moreover, the t_ind_ and slope of the time–concentration profile were not statistically significantly different (*p* > 0.05) between PVP K15, K30, K60 and K120 at the same concentrations. Finally, supersaturation studies in isoviscous solutions of PVP confirmed that solution viscosity has a negligible influence on the t_ind_ and the slope of the time–concentration profile of the drugs. In conclusion, this study showed that an increasing concentration of PVP in solution increases the onset time of nucleation and decreases the precipitation rate of the drugs, which can be explained by molecular interactions between the drug and polymer. In contrast, no significant influence of the viscosity of the dissolution media was seen for the onset of nucleation and the precipitation rate of the drugs, meaning that the molecular mobility of the drug in solution has no influence on the precipitation inhibition of the drug.

## Figures and Tables

**Table 1:** Viscosity of isoviscous solutions of PVP K15, K30, K60 and K120.

	K15	K30	K60	K120
Concentration (% (*w*/*v*))	3.13	2.81	0.33	0.25
Viscosity (mPa.s) mean ± SD	0.98 ± 0.03	1.05 ± 0.12	1.05 ± 0.12	1.04 ± 0.04

**Figure A1:**
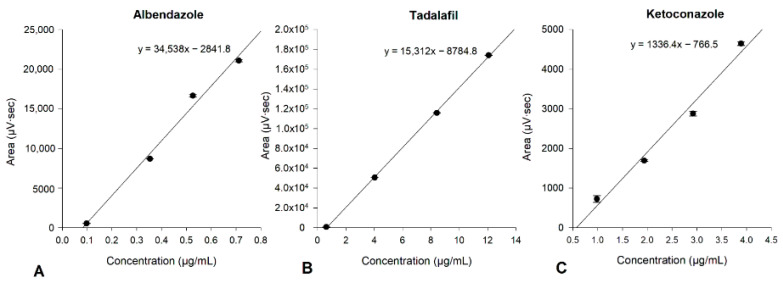
Standard curves for determination of the equilibrium solubility of the albendazole R^2^ = 0.997 (**A**), tadalafil R^2^ = 0.999 (**B**) and ketoconazole R^2^ = 0.989 (**C**) (*n* = 3) (mean ± SD).

**Figure A2:**
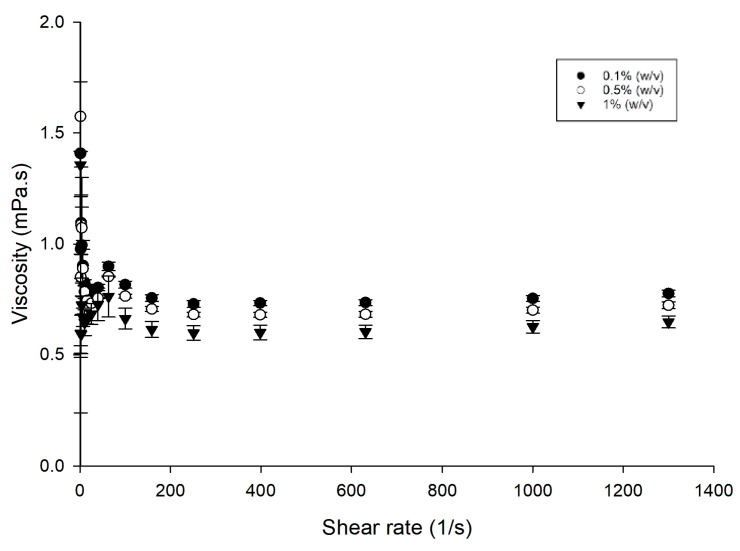
Viscograms of the solutions of PVP K15 at the concentrations of 0.1, 0.5 and 1% (*w*/*v*) (*n* = 3) (mean ± SD).

**Figure A3:**
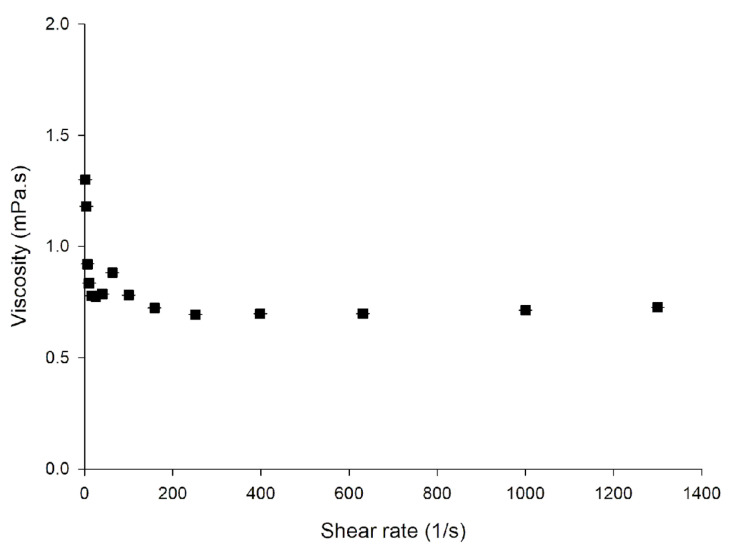
Viscogram of water (*n* = 3) (mean ± SD).

**Figure A4:**
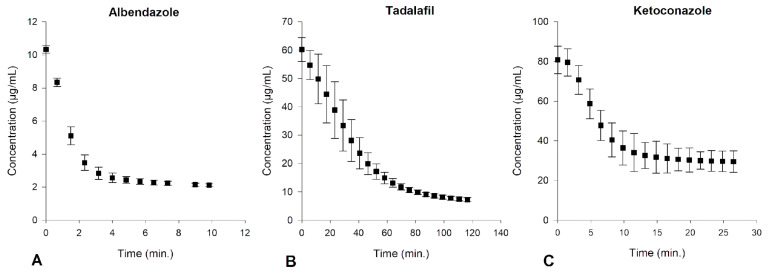
Time–concentration profiles of supersaturated solutions of albendazole (**A**), tadalafil (**B**) and ketoconazole (**C**) in phosphate buffer (*n* = 8) (mean ± SD).

## Data Availability

Data are contained within the article.
